# Primary solitary fibrous tumor of the stomach: A case report and a review of literature

**DOI:** 10.1097/MD.0000000000041096

**Published:** 2025-01-10

**Authors:** Ling-Yun Dong, Ying-Chun Li, Hai-Chao Tong, Yao-xing Guo, Le-Yao Li, Wan-Lin Zhang, Shuang Ma, Lian-He Yang, Philip Petersen, Endi Wang

**Affiliations:** a Department of Pathology, First Affiliated Hospital and College of Basic Medical Sciences, China Medical University, Shenyang, Liaoning, China; b Department of Pathology, Yangzhou Maternal and Child Health Hospital, Yangzhou, Jiangsu, China; c Department of Pathology, General Hospital of Northern Theater Command, Shenyang, Liaoning, China; d Department of Neurology, Sheng Jing Hospital of China Medical University, Shenyang, Liaoning, China; e Department of Pathology, Keck School of Medicine, University of Southern California, Los Angeles, CA.

**Keywords:** immunohistochemistry, solitary fibrous tumor, stomach neoplasms

## Abstract

**Rationale:**

Solitary fibrous tumors (SFTs) are spindle cell tumors that typically occur in the pleura and peritoneum, but very rarely in the stomach. To our best knowledge, there are only 10 cases reported in English literature. We reported a case of primary stomach SFT and summarized the characteristics of all previous cases, suggesting that pathologists and surgeons should include this disease in the differential diagnosis list of primary mesenchymal tumor of the stomach.

**Patient concerns:**

The patient suffered from epigastric pain for 5 months. Gastroscopy revealed a submucosa mass along the greater curvature centered on the gastric body. A repeat gastroscopy after 6 months showed a slight increase in mass size.

**Diagnoses:**

Endoscopic ultrasound showed the mass in the middle and lower portion of the stomach body, measuring 3.1cm in diameter, with a smooth surface. The patient was initially diagnosed with a gastrointestinal stromal tumor by gastroscopy. However, the pathologic morphology and immunohistochemical staining of resected specimens after surgery support a diagnosis of primary gastric SFT.

**Interventions:**

The patient underwent a laparoscopic gastric mass resection.

**Outcomes:**

The patient returned to hospital after 3 months with no recurrence or postoperative complications. During the 18-month follow-up period, the patient did not experience any tumor recurrence or metastasis.

**Lessons:**

This case teaches us that SFT should be included in the differential diagnoses of gastric primary spindle cell tumors, even though it is very rare in the stomach.

## 1. Introduction

Solitary fibrous tumor (SFT) is a rare mesenchymal neoplasm, often originating from the pleura, which was first described in 1931.^[1]^ It accounts for <2% of all soft-tissue tumors and can originate in nonpleural sites, including the peritoneum, pericardium, mediastinum, retroperitoneum, paranasal sinuses, nose, upper respiratory tract, and extrapleural soft tissue.^[2,3]^ SFT commonly affects adults without any sex predominance. Although it can develop at any age, the median age of diagnosis is 50, and there is no correlation between age and anatomic location.^[4–7]^ SFT can exist in numerous extrathoracic locations, including the meninges, orbit, peritoneum, breast, liver and urogenital organs. However, primary gastric SFT is extremely rare. To the best of our knowledge, only 10 cases of gastric SFT have been reported in the literature (Table 1).^[2,4,5,8–14]^ In this study, we present a 67-year-old Asian male with primary gastric SFT.

**Table 1:** Literature review of case reports of solitary fibrous tumor (SFT) in the stomach.

Case	Publication year	Age	Gender	Size (cm)	Clinical manifestation	Treatment
Shidham et al^[8]^	1997	77	Female	3	Asymptomatic	NA
Lee et al^[9]^	2004	70	Male	8.5 × 7 × 6	Upper abdominal cramp	Surgical wedge resection
Park et al^[2]^	2007	26	Male	5.4 × 5.2 × 4	Melena	Laparoscopic wedge resection
Nabeshima et al^[10]^	2015	43	Female	2.7 × 2 × 1. 5	Melena	LECS for wedge resection
Boskovic et al^[4]^	2015	65	Female	2.5 × 2.3 × 1	Asymptomatic	Surgical resection
Inayat et al^[5]^	2016	55	Female	0.71 × 0.67	Dyspepsia	Endoscopic resection
Xiang et al^[11]^	2016	56	Male	3 × 4.5	Melena	LECS
Voth et al^12]^	2018	68	Male	16 × 9	Flank pain	En bloc resection of the tumor along with partial gastrectomy involving the lesser curvature of the stomach
Kimmel et al^[13]^	2019	81	Female	7.5	Melena	Sleeve gastrectomy
Ababneh et al^[14]^	2020	79	Female	6.6	Asymptomatic	NA
Current report	2024	67	Male	3.11 × 1.72	Epigastric pain	Laparoscopic gastric mass resection

LECS = laparoscopic and endoscopic cooperative surgery, NA = not available, SFT = solitary fibrous tumor.

## 2. Case presentation

A 67-year-old man suffered from epigastric pain for 5 months. In June 2022, he visited a local hospital and underwent a gastroscopy. The procedure revealed a 3.0 × 2.5 × 2.5 cm^3^ mucosal bulge centered on the gastric body along the greater curvature. The mucosa of the fundus, gastric body, gastric horn, and antrum was pink to red, with a submucosal vascular network visible throughout. The gastric antrum showed multiple flaky areas of hyperemia. The pylorus exhibited good opening and closing movement, and the duodenal bulbous cavity was not deformed. He was diagnosed as gastrointestinal stromal tumor (GIST), esophagitis, and atrophic gastritis by gastroscopy. The patient denied any history of tumors.

**Figure 1.**
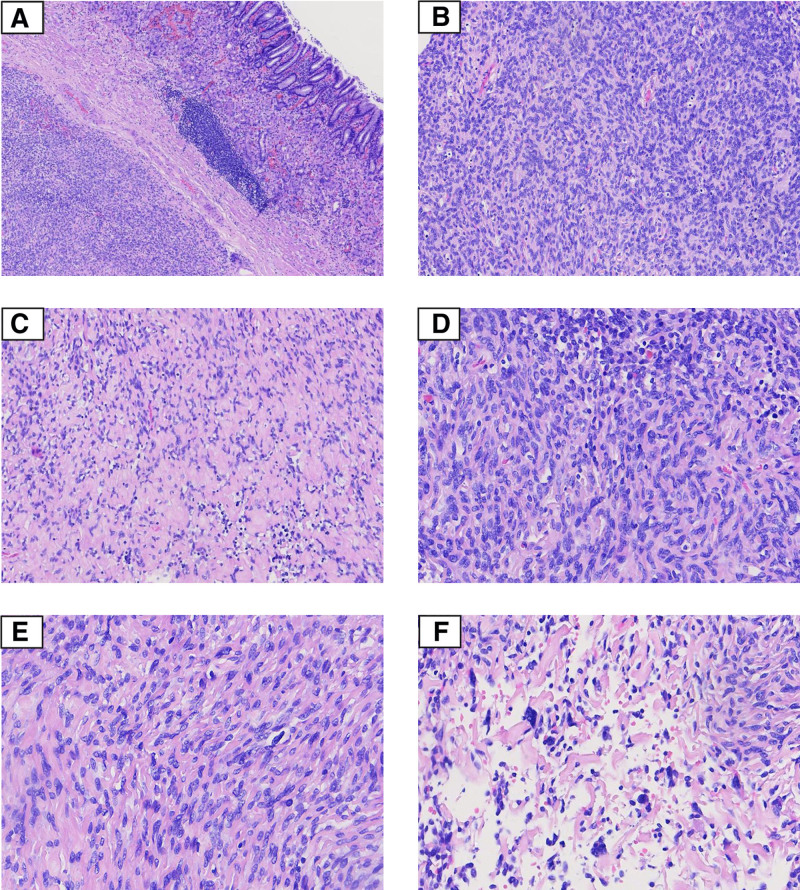
(A) Tumor cells are located under the gastric mucosa with clear boundaries (40×); (B) Tumor cells are densely arranged in bundles or swirls (100×); (C) Areas with sparse tumor cells showed interstitial hyaline degeneration (100×); (D) Tumor cells showed mild morphology with an oval nucleus and occasionally visible small nucleoli (200×); (E)Tumor cells arranged in a flowing water pattern with interstitial hyaline degeneration. Mototic figures are rare (200×); (F) Scattered multinucleated giant cells are observed in the interstitial hyaline degeneration (200×).

In November 2022, he visited our hospital for further treatment. An endoscopic ultrasound showed a bulge in the middle and lower portion of the gastric body, measuring 3.1 cm in diameter with a smooth surface. The mass had a clear border without erosion or ulcer formation. The patient completed relevant examinations and preoperative preparations, then underwent laparoscopic gastrectomy. Grossly, the tumor had a clear boundary with surrounding tissue, a gray to yellow surface, and a firm texture. The cut surface was pale with multifocal yellowish, gritty, stony-hard nodules and cystic changes. Microscopically, tumor cells were located under the gastric mucosa with a clear boundary (Fig. 1A). Tumor cells are relatively dense and arranged in bundles or vortices in some area. In other areas, the tumor cells were sparsely arranged and accompanied by significant interstitial hyaline degeneration (Fig. 1B, C). Under higher magnification, tumor cells exhibited indistinct borders, consistent sizes, a mild morphology, an oval nuclei, and an occasional small nucleoli (Fig. 1D, E). In some areas, hyaline degeneration of the interstitium could be found in the tumor tissue, with multinucleated tumor giant cells scattered among the hyalinized collagen fibers (Fig. 1F).

**Figure 2.**
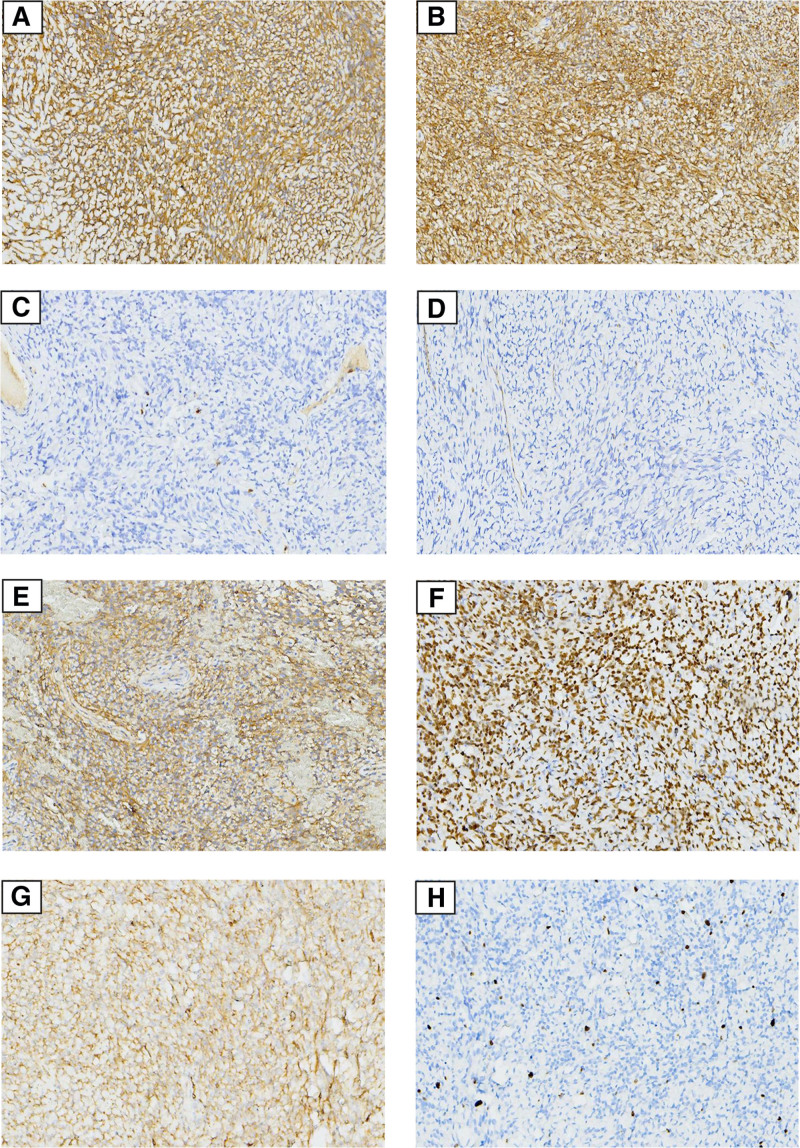
(A) Vimentin showed positive expression (100×); (B) Bcl-2 showed positive expression (C) CD117 showed negative expression (100×); (D) Dog-1 showed negative expression (100×); (100×); (E) CD99 showed positive expression (100×); (F) STAT-6 showed positive expression (100×); (G) CD34 showed positive expression (100×); (H) The Ki-67 proliferation index is approximately 2% (100×).

Immunohistochemical stains are positive for vimentin, CD34, CD99, Bcl-2, and STAT-6, but negative for, CK, S100, CD117, Dog-1, SMA, and β-catenin. The Ki-67 proliferation index was approximately 2% (Fig. 2). Both morphological and immunohistochemical studies suggest a diagnosis of primary gastric SFT. The patient returned to the hospital 3 months postoperation without any signs of recurrence or complications. During the 18-month follow-up period, the patient did not experience any tumor recurrence or metastasis.

## 3. Discussion

The pleura is the most common location of a SFT, and it was initially believed to originate from the mesothelium. While SFTs are well recognized in the pleura, diagnosing a SFT in the stomach can be challenging.^[15–19]^ The symptoms of a SFT are usually nonspecific and vary depending on the lesions. For serological laboratory testing, tumor markers are neither specific nor sensitive for SFT. A high expression of serum insulin-like growth factor-II has been reported in patients presenting with hypoglycemia.^[20,21]^

Pathological studies are crucial for confirming the diagnosis. The morphology of SFT varies and primarily depends on the relative ratio of tumor cells to fibrous and mesenchymal elements. Features include ovoid to fusiform spindle cells with indistinct cell borders arranged in a patternless pattern, dilated staghorn-like vasculature, and hyalinized to collagenous stroma. Sometimes, morphologically benign fibroblasts are distributed between collagen. In rare cases, interstitial mucoid degeneration and a lipomatous variant may occur.

Immunohistochemistry and comprehensive ultrastructural research have led to the current understanding that SFTs originate from primitive fibroblast-like cells. A frequent inversion within the same arm of chromosome12q13 has been observed in recent studies of SFT. This inversion results in a chromosomal rearrangement and fusion of the neighboring genes NGFI-A-binding protein2 and STAT6, which may be the initial pathological mechanism for the occurrence and development of SFT.^[15–21]^ This gene fusion triggers an overexpression of STAT6 protein in the tumor nuclei. Studies have shown that the positive expression of the nuclear transcription factor STAT6, as revealed by immunohistochemical staining, is highly sensitive and specific for diagnosing SFT. It also shows high consistency with the NGFI-A-binding protein2–STAT6 fusion gene.^[22]^ A small number of SFT cases can exhibit negative immunohistochemical STAT6 expression. In such instances, GRIA2 testing enhances the diagnostic sensitivity and specificity for SFT. Immunohistochemistry aids diagnosis due to histologic variability, especially for tumors in uncommon locations, as was the case with our patient. In addition to STAT6, most cases express GRIA2, CD34, Bcl-2 and CD99. Malignant SFT is characterized by infiltrating boundaries, pleomorphism, abundant cells, mitotic figures >4/10 HPF, and necrosis.

The diagnosis of gastric SFTs should be differentiated from gastrointestinal stromal tumors, gastric schwannomas, and leiomyomas. The tumor cells in GISTs are typically spindle or epithelioid cells. Spindle cells are commonly arrange in bundles, swirls, or palisades. Epithelioid cells appear round or polygonal, with clearly demarcated cell membranes, a clear to eosinophilic cytoplasm, and a central nucleus. The most important diagnostic clue for GISTs is the positive expression of CD34, CD117 and Dog-1.

Gastric schwannomas are histologically diverse tumors, containing long spindle-shaped cells, arranged in long curved or interwoven bundles interlacing with collagen. There is often nuclear palisading, hyalinized vessels, and Verocay bodies. The tumor cells exhibit varying degrees of cellular atypia and mitoses. They express S-100 protein, NSE, and SOX-10, but are negative for STAT6, CD34, CD99, and Bcl-2.

Gastric leiomyoma tumor cells have a typical rod-shaped nucleus and a rich eosinophilic cytoplasm with thick-walled large blood vessels. The tumor cells and muscularis mucosae are closely intertwined and the tumor forms a bundle-like to braided pattern. The cytoplasm exhibits a deep eosinophilic color, and the ends of the nucleus are blunt and round. Leiomyosarcoma tumor cells have a bundle-like arrangement, with mitoses, necrosis, and an infiltrative growth pattern. Both are positive for SMA, Calponin, H-Caldesmon, and desmin, while CD34 and STAT6 are negative.

Overall, solitary fibrous tumors of the stomach are exceedingly rare. Symptoms of stomach SFT are usually nonspecific and vary depending on the lesion, their imaging and pathological features are also easily confused with those of primary mesenchymal tumors of the stomach, which make it difficult to diagnose. Primary mesenchymal tumors of the stomach, including stromal tumors, leiomyomas, fibromatosis, schwannomas and so on, while SFTs are easily overlooked due to their rare incidence rate. However, SFTs specifically express nuclear STAT-6, so we believe that the diagnosis of primary stomach SFTs relies on pathologists conducting a comprehensive analysis of immunohistochemical indicators.

## Acknowledgments

This study was supported by the “Supporting the high-quality development of science and technology funding projects in China Medical University” (2023020778-JH2/202, to L.-H. Yang), National Natural Science Foundation of China (Grant No. 81301930 to L.-H. Yang), General project fund of the Education Department of Liaoning Province (Grant No. L2015595 to L.-H. Yang), Key R&D Program Projects of Liaoning Province (Grant No. 2018225085 to L.-H. Yang), Natural Science Fund of Liaoning Province (Grant No. 2019JH3/10300420 and 2019-MS-374 to L.-H. Yang), Natural Science Fund of Liaoning Province (Grant No. 2020-MS-142 to S. Ma), Natural Fund Guidance Plan of Liaoning Provincial Science and Technology Department (Grant No.2019-ZD-0735 to S. Ma), and 345 Talent Project of Shengjing Hospital of China Medical University (Grant No. M0364 to S. Ma).

## Author contributions

**Conceptualization:** Ling-Yun Dong, Hai-Chao Tong.

**Data curation:** Ying-Chun Li, Hai-Chao Tong.

**Formal analysis:** Ling-Yun Dong, Shuang Ma.

**Funding acquisition:** Lian-He Yang.

**Investigation:** Ying-Chun Li, Shuang Ma.

**Methodology:** Lian-He Yang.

**Project administration:** Lian-He Yang.

**Resources:** Yao-xing Guo, Le-Yao Li.

**Software:** Yao-xing Guo, Le-Yao Li.

**Supervision:** Endi Wang.

**Validation:** Shuang Ma, Lian-He Yang.

**Visualization:** Wan-Lin Zhang.

**Writing – original draft:** Ling-Yun Dong.

**Writing – review & editing:** Lian-He Yang, Philip Petersen.
